# Less Expectation, Less Pain: Low Wealth Alleviates Sense of Unfairness

**DOI:** 10.3389/fpsyg.2021.571952

**Published:** 2021-04-28

**Authors:** Guanxiong Pei, Jia Jin, Taihao Li, Cheng Fang

**Affiliations:** ^1^Research Center for Advanced AI Theory, Zhejiang Lab, Hangzhou, China; ^2^Laboratory of Applied Brain and Cognitive Sciences, School of Business and Management, Shanghai International Studies University, Shanghai, China; ^3^Accounting Department, Zhejiang Radio and TV Group, Hangzhou, China

**Keywords:** fairness, objective wealth, ultimatum game, feedback-related negativity, late frontal negativity, event-related potentials

## Abstract

Objective wealth plays an important role in social interaction and economic decision making. Previous studies indicate that objective wealth of others may influence the way we participate in resources allocation. However, the effect of objective wealth on responses to fairness-related resource distribution is far from clear, as are the underlying neural processes. To address this issue, we dynamically manipulated proposers’ objective wealth and analyzed participants’ behavior as responders in a modified Ultimatum Game, during which event-related potentials were recorded. Behavioral results showed that participants were prone to reject unfair proposals although that rejection would reduce their own benefit. Importantly, participants were more likely to accept unfair offers from proposers with low objective wealth than from proposers with high objective wealth, with a drastic increase in acceptance rates of unfair offers from 32.79 to 50.59%. Further electrophysiological results showed that there was significantly enhanced feedback-related negativity amplitude toward proposers with high (relative to low) objective wealth for unfair offers. Furthermore, the late frontal negativity amplitude was larger for all the conditions which are not high-fair, which might be the only option that did not elicit any ambiguity. These findings suggest a strong role of proposers’ objective wealth in modulating responders’ behavioral and neural responses to fairness.

## Introduction

Ultimatum Game (UG) is a primary experimental tool used to investigate the underlying mechanisms of human fairness (Güth et al., 1982; Thaler, 1988; Camerer and Thaler, 1995). In a typical UG, one player acts as the proposer and is given a sum of money to split between himself or herself and the second player, who acts as a responder. The responder has to decide whether to accept or reject the offer. If the responder accepts the offer, each player gets the proposed share. If the responder rejects the offer, both players come away with nothing (Güth et al., 1982). Traditional economic theory expects people to be rational and self-interest driven, which means that the proposer should offer the smallest amount possible and that the responder should accept any non-zero offer. However, in reality, players do not follow this prediction at all. Proposers typically offer nearly 40–50% of the total money, and responders generally reject offers of 30% or less (Güth et al., 1982; Güth and Tietz, 1990; Bolton and Zwick, 1995; Nowak et al., 2000). This “irrational” behavior is explained by human beings’ preference for a fairness norm (Güth et al., 1982; Polezzi et al., 2008; Hu et al., 2014; Fortin et al., 2019; Nai et al., 2020).

As an essential social norm, fairness considerations are influenced by numerous factors, such as perceived intention (Ma et al., 2015a), social distance (Campanhã et al., 2011), facial expression (Mussel et al., 2014), facial attractiveness (Ma et al., 2015b), and transparency in organizational decisions (Nai et al., 2020). One other critical factor is the objective wealth of the interacting persons (Kan Holm and Engseld, 2005; De Cremer, 2007; Ahl and Dunham, 2019). Objective wealth is defined as the availability of economic resources, absolute income and the value of one’s assets holdings (Howell and Howell, 2008; Kaiser et al., 2017). As an important factor in economic decision making, personal objective wealth is closely related with almost all aspects of our life, such as investment in the public good (Conroy and Emerson, 2014), prosocial behaviors (Penner et al., 2005; Piff et al., 2010), mental health (Lee et al., 2013), perceived individual and collective efficacy (Fernández Ballesteros et al., 2002), human mate choice (Bereczkei et al., 1997), life satisfaction and subjective well-being (Johnson and Krueger, 2006), and social relationship (Ranta et al., 2019).

Previous studies have demonstrated that others’ objective wealth may influence the way we participate in wealth allocation and the process of fairness consideration (Kan Holm and Engseld, 2005; De Cremer, 2007; Paulus, 2014; Rodrigues et al., 2015; Ahl and Dunham, 2019). During a UG task, proposers preferred to give higher offers to responders with low income rather than ones with high income (Kan Holm and Engseld, 2005). It was also indicated that participants tend to equalize their earnings with the poorer participants (De Cremer, 2007; Ahl and Dunham, 2019). In a DG task, there is no opportunity for the receiver to bargain with the allocator, but the allocator usually still shares part of the money with the receiver, under the pressure of social norm of fairness. More importantly, they were more generous to people with less income (Rodrigues et al., 2015). Even children would give more resource to poor individuals than wealthy ones during a resource allocation game (Paulus, 2014). This provides an optimistic picture of human behavior.

However, most studies focused on how proposers might perceive objective wealth of responders in the bargaining, few of them shed light on the effect of proposers’ objective wealth during responders’ decision making. In this study, we employed a modified version of the UG in which participant acted as responders and were informed of proposers’ objective wealth before receiving offers. In each trial, a priming stimulus (photo of the proposer and gold-coin-like cues indicating his/her objective wealth) was added before the offer presentation. Thus, participants acting as responders in the UG can make inferences regarding the proposers’ objective wealth. The objective wealth of proposers and the fairness of the offers in the UG were varied systematically over different trials of the game. Event-related potentials (ERPs) were record time-locked to the priming stimulus and to the offer presentation. The purpose of the current study is to examine how the brain responds to fair and unfair offers in the UG and, more importantly, to explore how the perceived objective wealth modulates the responder’s brain responses to different offers.

Behaviorally, we focused on the acceptance rate of UG offers. We hypothesized that the acceptance rate would decline as the level of fairness decreased. Specifically, we expected that when participants faced with the poor, compared with the rich, they would recognize the need of people with less income and have less expectation to fair offers and, as a result, be more likely to accept unfair offers. At the neural level, we focused specifically on the feedback-related negativity (FRN) and late frontal negativity (LFN) responses to offers.

According to previous studies, in the UG, one psychophysiological marker that has often been used to link brain activation to behavior is FRN (Miltner et al., 1997; Rodrigues et al., 2015). FRN is a frontal-central negative deflection peaking at 200–350 ms after feedback presentation (Hajcak et al., 2006; Meng and Ma, 2015). From fMRI evidence, FRN is thought to be generated in the anterior cingulate cortex (ACC) (Ma et al., 2015a; Rushworth and Behrens, 2008). It was suggested that the involvement of ACC in UG task were associated with detecting and responding to violation of fairness-related norms (Sanfey et al., 2003; Montague and Lohrenz, 2007). Reinforcement learning theory is a popular theory of FRN in reflecting the outcome evaluation process. In terms of reinforcement learning theory, larger FRN values would be induced by unexpected feedback (Harris and Fiske, 2010; San Martín, 2012; Chandrakumar et al., 2018; Yaple et al., 2018). Ma et al. (2014) showed that effort strengthened the expectation to good results and that violation of this larger expectation induced a more negative FRN. Furthermore, violation of social norms can also produce an enhanced FRN in economic interactions. It has been demonstrated that an unfair offer can induce a more negative FRN compared to fair offers in wealth allocation (Polezzi et al., 2008; Boksem and De Cremer, 2010; Jin et al., 2020). Similarly, in this study, we postulate that unfair offers would elicit a more negative FRN compared with fair offers. In addition, low objective wealth is always connected with the need for money. Participants may recognize the need of people with low income and feel compassion for them. Thus, they may have lower expectations of the offers from proposers with low objective wealth. In contrast, participants might have a relatively higher expectation of fair offers from proposers with high objective wealth. When this expectation is violated, a stronger prediction error might occur, which might elicit a larger FRN deflection.

Another component is the LFN, a negative component reaching peak amplitude around 400 ms post stimulus onset over frontal-central areas, which can index the degree of ambiguity or conflict (Astle et al., 2006; Mueller et al., 2007; Polezzi et al., 2008; Civai et al., 2020; Wagner-Altendorf et al., 2020). In an object recognition task, Schendan and Kutas (2003) found that LFN was larger for those conditions in which objects were harder to identify. Polezzi et al. (2008) found that mid-value offers gave rise to LFN compared to fair and unfair offers. The mid-value offers in UG appeared to induce conflicting responses tendencies (i.e., acceptance and rejection), therefore making the decision more difficult and eliciting a higher potential. Furthermore, it was demonstrated that the amplitude of LFN was directly linked to the ambiguity of the violation of fairness in a third-party altruistic punishment game. Larger negativity reflected the increased difficulty of processing the degree of ambiguity and the need to integrate more information before responding (Civai et al., 2020). Given that LFN is a reflection of ambiguity or conflict, we speculate that LFN would become larger in unfair conditions, especially to the violation of social norms by proposers with high objective wealth. People with high objective wealth often live a better life and have preferential access to resources vital for survival, including food, information, land, and power. Previous studies showed that people with high objective wealth were more generous and more likely to engage in prosocial behaviors (Penner et al., 2005; Piff et al., 2010). Thus, the unfair offers by proposers with high objective wealth might cause greater conflict.

The present study was designed to examine the modulation role of proposers’ objective wealth on responders’ behavioral and neural responses to fairness. We expected that participants would have higher expectations of proposers with high objective wealth, with violations giving rise to higher rejection rates and enhanced FRN amplitudes. In addition, participants would show increased neural salience and cognitive conflict in response to unfair offers by proposers with high objective wealth.

## Materials and Methods

### Participants

Eighteen healthy, right-handed college students (nine males and nine females) aged 20–26 years (*M* = 23.17 years, SD = 1.58 years) participated in this experiment. They had normal or corrected-to-normal vision and did not have a history of mental or neurological disease. Written informed consent was obtained from all subjects according to the procedure approved by the institutional Internal Review Board (IRB). Data from one subject was removed because of excessive recording artifacts, leaving 17 valid subjects for final analysis. All subjects volunteered to join in this experiment. They were paid 30 Chinese Yuan (about $4.25) as basic payment and were informed that additional monetary rewards would be paid according to the income from two randomly selected trials in the UG.

### Materials

The stimuli were half-length Asian photos obtained from the Internet. All of them were edited to a uniform size (2.03 by 2.54 cm, 240 by 300 pixels) and gray-processed using Adobe Photoshop software (Adobe Systems Incorporated, San Jose, CA, United States) to ensure consistency in the background, brightness, and color saturation. In previous studies, it was demonstrated that the objective wealth (e.g., income, savings, and assets) of others could usually be inferred from clothing and physical appearance (Turner, 2001; de Jong et al., 2008). Twenty participants who did not participate in the electrophysiological experiment rated the perceived objective wealth of characters in 90 photos through a five-point Likert scale ranging from 1 (very high objective wealth) to 5 (very low objective wealth). Concerned about the equal balance of gender and age among the faces of the proposers, 20 photos with high ratings and 20 photos with low ratings were selected and classified as the high objective wealth group and the low objective wealth group, respectively. The ratings of the two groups were evaluated with a paired *t*-test. The objective wealth was significantly different between the two categories [high (*M* = 4.51, SD = 0.970); low (*M* = 2.06, SD = 0.442); *t*(19) = −31.132, *p* < 0.001]. What’s more, the objective wealth information was further conveyed through a set of coins. In the high objective wealth group, four or five filled coins were presented beneath the corresponding half-length photos. In the low objective wealth group, one filled coin or two filled coins were presented.

### Design and Procedure

Participants were comfortably seated in a dim, sound-attenuated, and electrically shielded room. E-Prime 2.0 software (Psychology Software Tools, Pittsburgh, PA, United States) was used for presenting stimuli and triggers. The experimental stimuli were presented at the center of a computer screen at a distance of 100 cm, with a visual angle of 8.69° × 6.52° (15.2 cm × 11.4 cm, width × height). The experiment had a 2 × 2 within-participant factorial design, with the first factor referring to the level of objective wealth (high vs. low) and the second factor referring to the fairness (fair vs. unfair).

Participants were instructed about the rule of the UG on paper handouts. To increase the credibility of the task, participants were told that they were part of a lager ongoing study involving different age groups, in which they would play only the role of the responder. Participants were told that they would see offers from different proposers on a computer screen. They were instructed to use the keypad to make their choices (assignment of accept/reject keys was counterbalanced across participants). All participants were informed that both themselves and the proposers would be paid according to the choices made by them. To further increase credibility, participants were informed that the photos of proposers they would see during the task were from persons who had submitted their offers previously and would not be present at the time of the experiment. Photo priming is beneficial to increase the interactivity of the whole experiment and helpful to gain insights into how people make decisions in real-life situations (Zaatari et al., 2009; Forgas and Tan, 2013; Ma et al., 2015b). Gold-coin-like cues paired with the photos were information about the objective wealth of them. Actually, there were no real proposers, gold-coin-like cues were designed to frame the objective wealth, and the offers were systematically made up by us prior to the experiment. Furthermore, they were told that they would have the chance to play the role of proposer with persons who would participate in the future. They were asked if they would allow their photos and the information about their personal objective wealth to be taken and used in future sessions. If they agreed, their photograph was taken, and they were asked to fill out questionnaire concerning about their personal demographic and socioeconomic information. Seven participants declined having their photograph and information taken.

**FIGURE 1:**
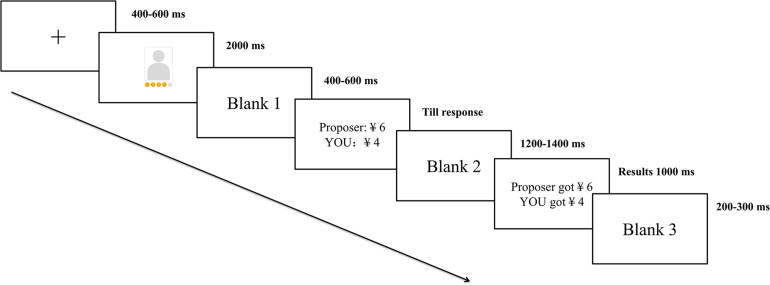
Sequence of events in a single trial.

Prior to the formal experiment, each participant practiced for five trials to become familiar with the experimental procedure. The formal experiment consisted of four blocks, each containing 45 trials. All of the trails in a block were presented in a random order. A single trial is illustrated in Figure 1. The trial commenced with a fixation that lasted for 400–600 ms. A photo of the proposer and objective wealth indicators (gold-coin-like cues) were subsequently presented for 2000 ms. After a random blank screen that lasted for 400–600 ms, the proposal of how to split ¥10 was shown. The participant was asked to decide whether to accept or reject the proposal by pressing the keypad. If the participant accepted the proposal, ¥10 would be split as the proposer suggested; otherwise, both the proposer and the participant would receive no compensation in that round. The stake size is consistent with previous studies which use ultimatum game as an experimental tool to investigate the underlying mechanisms of human fairness (e.g., Wu et al., 2011; Zhou and Wu, 2011; Hu et al., 2014). After another random blank screen to separate sequential stimuli, lasting 1200–1400 ms, the final income from this trial was presented for 1000 ms. The inter-trial interval lasted 200–300 ms. There were five different offer conditions (¥1, ¥2, ¥3, ¥4, and ¥5). An average analysis was based on the trials that represented unfair offers (¥1 and ¥2) and fair offers (¥4 and ¥5), repeated 40 times separately. In addition, another 20 trials with an offer of ¥3 were used as fillers. The condition in which responders received offers of more money than the proposers was not included because previous behavioral studies have shown that, even in situations in which proposers and responders have close relationships, no more than 52% of the total was provided by proposers to responders (Burnham, 2003; Charness and Gneezy, 2008; Wu et al., 2011).

### EEG Recordings and Analysis

Electroencephalograms (EEGs) were continuously recorded from 64 scalp sites (band-pass 0.05–70 Hz, sampling rate 500 Hz) with a Neuroscan Synamp2 Amplifier (Scan 4.3.1; Neurosoft Labs Inc., VA, United States). A cephalic (forehead) location was used as a ground. The left mastoid served as an on-line reference, and recorded EEGs were off-line re-referenced to the average of the left and right mastoids. Vertical and horizontal electro-oculograms (EOG) were recorded with two pairs of electrodes, one pair placed above and below the left eye and another pair 10 mm from the lateral canthi. Electrode impedance was kept below 5 kΩ during the recording.

In the offline EEG analysis, ocular artifacts were removed followed by digital filtering through a zero phase shift (low pass at 30 Hz, 24 dB/octave). EEGs epochs of 1000 ms (with a 200-ms pre-stimulus baseline) were extracted offline. The whole epoch was subsequently baseline-corrected using the 200 ms interval prior to stimulus onset. Single trial data were corrected for vertical and horizontal eye movements using a correlative eye movement algorithm (Miller et al., 1988). Trials containing bursts of electromyography activity, amplifier clipping, or peak-to-peak deflections exceeding ±80 μV were excluded. During the offer presentation, the EEG epochs were separately averaged for objective wealth (high/low) × fairness (fair/unfair offer). Therefore, there were four conditions: high-fair, high-unfair, low-fair, and low-unfair. All these conditions encompassed a minimum of 30 valid trials.

Considering the fronto-central distribution of the FRN (San Martín, 2012; Meng and Ma, 2015) and the topographic map, data from the electrode sites F3, Fz, F4, Fc3, FCz, and Fc4 were analyzed. The ERP waveforms of FRN were averaged across the electrode cluster. Because the most negative peak of the FRN appeared approximately 320 ms after the onset of the offer, mean amplitudes in the 290–350 ms time window were analyzed using a 2 (objective wealth) × 2 (fairness) repeated-measures ANOVA. Given that the maximum LFN amplitudes were observed at frontal sites, data from the electrode sites F3, Fz, F4, Fc3, FCz, and Fc4 were analyzed. ERP waveforms of LFN were averaged across the electrode cluster. Because the most negative peak appeared approximately 450 ms after the onset of the offer, mean amplitudes in the time window of 400–500 ms were calculated, and ANOVA was conducted for two within-subject factors: objective wealth and fairness. Simple effect analysis was conducted when the interaction effect achieved significance. The Greenhouse–Geisser correction was applied in all statistical analyses when necessary (Greenhouse and Geisser, 1959).

## Results

### Behavioral Performance

**FIGURE 2:**
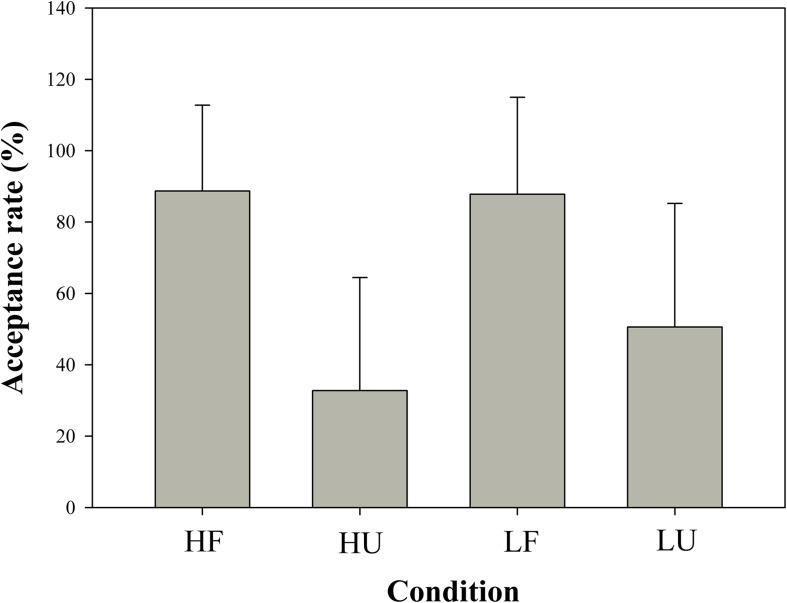
Acceptance rates for four conditions: “high objective wealth-fair” (HF), “high objective wealth-unfair” (HU), “low objective wealth-fair” (LF), “low objective wealth-unfair” (LU).

The acceptance rates for the different division schemes were 88.68% (high-fair, SD = 0.241), 32.79% (high-unfair, SD = 0.317), 87.79% (low-fair, SD = 0.272), and 50.59% (low-unfair, SD = 0.346), as shown in Figure 2. A 2 (objective wealth) × 2 (fairness) repeated-measures ANOVA revealed a main effect of fairness [*F*(1,16) = 23.936; *p* < 0.001; η^2^ = 0.599] but not of objective wealth [*F*(1,16) = 1.911; *p* = 0.186; η^2^ = 0.107]. The main effect of offer type suggested higher acceptance rates for fair offers than for unfair ones, which interacted with objective wealth [*F*(1,16) = 7.080; *p* = 0.017; η^2^ = 0.307]. We further examined the simple effect of objective wealth separately in fair and unfair conditions. The objective wealth is significant in the unfair condition [*t*(16) = −2.355; *p* = 0.032] but not in the fair condition [*t*(16) = 0.135; *p* = 0.894]. The acceptance rates are significantly higher to proposers with low objective wealth than to proposers with high objective wealth when the offer is unfair.

### ERPs

**FIGURE 3:**
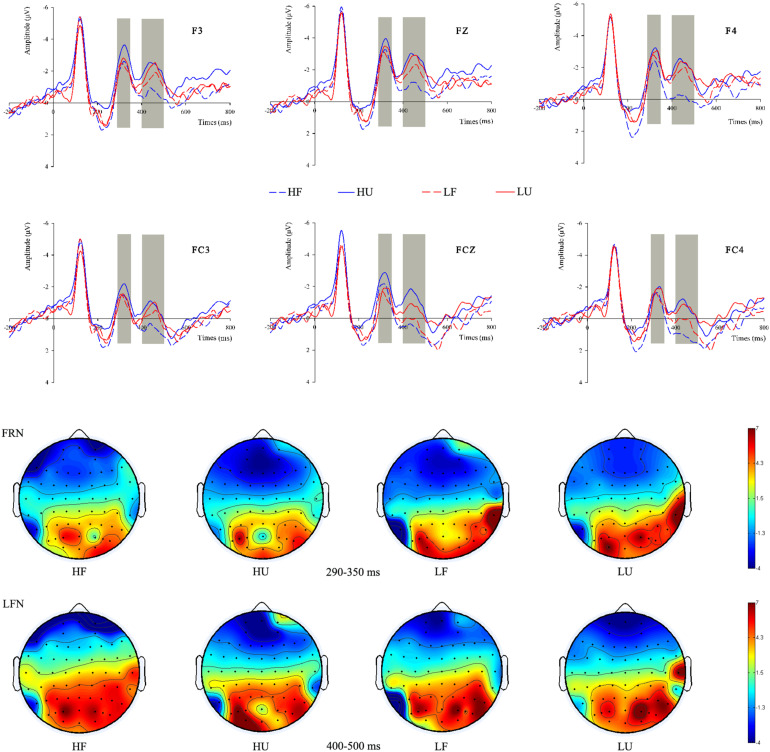
The ERP grand-average waveforms of FRN and LFN at F3, Fz, F4, FC3, FCz, and FC4 for four conditions. The shaded 290–350 ms time window was used for the mean FRN amplitude. The shaded 400–500 ms time window was used for the mean LFN amplitude. The scalp topographic distributions of the FRN and LFN were provided.

The evaluation of division schemes is mainly reflected in the FRN and the LFN. As presented in Figure 3, the mean FRN amplitudes in the 2 (objective wealth) × 2 (fairness) conditions are −1.439 μV (high-fair), −2.496 μV (high-unfair), −2.314 μV (low-fair), and −1.514 μV (low-unfair). ANOVA analysis for the FRN revealed no significant main effects of either objective wealth [*F*(1,16) = 0.018; *p* = 0.895; η^2^ = 0.001] or fairness [*F*(1,16) = 0.119; *p* = 0.734; η^2^ = 0.007]. However, the interaction effect of objective wealth and fairness is significant [*F*(1,16) = 21.363; *p* < 0.001; η^2^ = 0.572], with enhanced FRN amplitude toward proposers with high objective wealth compared with proposers with low objective wealth when the offer is unfair [*F*(1,16) = 4.659; *p* = 0.047; η^2^ = 0.291] but not when the offer is fair [*F*(1,16) = 3.965; *p* = 0.064; η^2^ = 0.248]. We further examined the fairness effect in the high and low objective wealth conditions, respectively; it is significant in the high objective wealth condition [*F*(1,16) = 5.398; *p* = 0.034; η^2^ = 0.337] but not in the low objective wealth condition [*F*(1,16) = 4.185; *p* = 0.057; η^2^ = 0.262].

As shown in Figure 3, the mean LFN amplitudes in the 2 (objective wealth) × 2 (fairness) conditions are 0.463 μV (high-fair), −1.305 μV (high-unfair), −0.582 μV (low-fair), −0.842 μV (low-unfair), respectively. The main effects of objective wealth [*F*(1,16) = 0.457; *p* = 0.509; η^2^ = 0.028] and fairness [*F*(1,16) = 4.321; *p* = 0.054; η^2^ = 0.213] failed to reach significance. However, the interaction effect of objective wealth and fairness is significant [*F*(1,16) = 6.323; *p* = 0.023; η^2^ = 0.283]. We examined the fairness effect in the high and low objective wealth conditions, respectively; it is significant in the high objective wealth condition [*F*(1,16) = 7.045; *p* = 0.017; η^2^ = 0.441] but not in the low objective wealth condition [*F*(1,16) = 0.320; *p* = 0.580; η^2^ = 0.020]. In addition, for proposers with low objective wealth in comparison with high financial capable proposers, LFN amplitude was enhanced for fair offers [*F*(1,16) = 5.045; *p* = 0.039; η^2^ = 0.316] and was not significantly different for unfair offers [*F*(1,16) = 0.643; *p* = 0.435; η^2^ = 0.040].

## Discussion

Fairness is one of the most fundamental aspects of social preferences, and it is deeply influenced by the perceived objective wealth of others. In this study, we explored how the perceived objective wealth of proposers influenced responders’ behaviors as well as their brain responses to various offers in social interactions. We revised the traditional UG by adding a proposer-photo priming step and adopted a 2 (high/low objective wealth) by 2 (fair/unfair offer) experimental design to have participants processing objective wealth and fairness simultaneously.

In this study, behavioral results showed that participants were prone to reject unfair proposals although that would reduce their own benefit. Compared with the fair offers, acceptance rates were significantly reduced when the offers were unfair. This is consistent with previous findings that people do not exclusively pursue material self-interest. They are also motivated by fairness considerations and care about social goals (Knoch et al., 2006; Van der Toorn and Jost, 2014). Violations of fairness are prone to elicit anger and a desire to restore justice (Haidt, 2003). People are willing to reject unfair distributions in micro-social transactions so that they can punish proposers for unfair treatment (Fehr and Schmidt, 1999; Ma et al., 2015b).

Furthermore, the results showed that the acceptance rates were modulated by the objective wealth of proposers. A comparison of acceptance rates indicated that responders were more likely to accept unfair offers from proposers with low objective wealth, with a drastic increase in the acceptance rate of unfair offers from 32.79 to 50.59%. Participants were more likely to accept the offer in the UG when they expected less (Hu et al., 2014). In the LU condition of the present study, participants might recognize the need of people with low income. This provided an acceptable explanation for the unfair proposals given by the poor, and participants might reduce requirements and expectations of those proposers. Combined with the sympathy to the poor, participants were willing to sacrifice part of the benefits, which led to a higher acceptance rate for unfair offers.

After the onset of the proposal, the FRN results showed that the interaction effect between objective wealth and fairness is significant. There was significantly enhanced FRN amplitude toward proposers with high objective wealth compared with proposers with low objective wealth for unfair offers but not for fair offers. This reflects the more important role of objective wealth consideration when an offer is unsatisfactory. In the UG, we speculate that fairness is the dominant factor in outcome evaluation. Participants did not care much about the objective wealth of proposers for fair proposals, as reflected by the absence of significant differences in FRN amplitudes between the high-fair (HF) and low-fair (LF) conditions. However, when the proposal is unfair, participants pay special attention to the objective wealth of proposers, as reflected in the significantly different FRN amplitudes between the HU and LU conditions. This demonstrated that in dynamic interactions, participants are not concerned only with objective outcomes. Instead, the objective wealth of proposers is an important concern.

In pioneering studies using the UG paradigm, perceived unfairness was reported to be moderated by many factors, such as facial attractiveness, facial expression, and perceived intention (Mussel et al., 2014; Ma et al., 2015a,b). Smiles, beautiful face, or perceived good intentions alleviate people’s perceived unfairness, as reflected in the FRN pattern. Similarly, low objective wealth also alleviates people’s sense of unfairness. Previous studies have shown that the need of others has a strong effect on fairness perception (Eckel and Grossman, 1996; Rodrigues et al., 2015). Low objective wealth is always connected with the need for money and other resources. In the LU condition of the present study, participants might recognize the need of people with low income. This provided an acceptable explanation for the unfair proposals given by the poor, and participants might reduce requirements and expectations of those proposers, leading to the reduced FRN to unfair offers from proposers with low objective wealth. In other words, low objective wealth can alleviate the social pain brought by unsatisfactory outcomes. People are inclined to tolerate unfair distributions from proposers with low objective wealth.

Furthermore, the fairness effect of FRN is significant in the high objective wealth condition but not in the low objective wealth condition. FRN reflects a reinforcement learning signal that occurs whenever outcomes are worse than expected (Harris and Fiske, 2010; San Martín, 2012). In the HU condition, as proposers with high objective wealth have greater access to resources, they were expected to give higher offers. The discrepancy between expected outcome and real outcome gave rise to larger FRN amplitude. People always have higher expectations of the rich than of the poor. It was demonstrated that teachers’ expectations for students from high-socioeconomic background would be higher than for students in other scenarios (Auwarter and Aruguete, 2008). People from high GDP nations are expected to show more prosocial behaviors (Kraus and Callaghan, 2016). Previous studies have also shown that high-income individuals are judged as more trustworthy than low-income individuals (Qi et al., 2018). Consistent with previous studies, participants withheld higher expectations and were more concerned with the fairness of proposed offers from the proposer with high objective wealth. In other words, the enhanced expectation toward fair offers might have strengthened fairness considerations in the high objective wealth condition.

A further distinction in processing the different types of offers was reflected in the LFN. In terms of LFN, previous studies mainly reported that its amplitude is closely related to ambiguity or conflict. Larger negativity reflected the increased difficulty of processing the degree of ambiguity (Astle et al., 2006; Mueller et al., 2007; Polezzi et al., 2008; Civai et al., 2020; Wagner-Altendorf et al., 2020). In the present study, when the rich proposed an unfair offer and were stingy, it might lead to a larger conflict with the social norm and cause the mismatch between a large amount of money owned and little money forgone. This may be the reason for the greater LFN in the HU condition compared with HF. Moreover, interestingly, in fair conditions, there is a larger LFN in response to the poor compared with the rich. People are in sympathy with those in poverty and are sensitive to the “need” of people. From a visual inspection of the images (Figure 3), LFN is larger for all the conditions which are not HF, which might be the only option that does not elicit any ambiguity or conflict in the participants (LF may elicit some ambiguity in that participants may feel that low-income proposers could have offered them less). It further indicated the modulatory role of objective wealth on fairness consideration.

Of course, the results reported here are restricted to a sample of students, which may threaten external validity. Firstly, college students share a particular personal economic background and financial status. It would be more interesting to extend the sample to include working adults, both wealthy and poor. Secondly, there is now plenty of evidence demonstrating that students are slightly less “pro-social” than other groups in a variety of designs and settings. For example, students have been shown to behave less generously (Carpenter et al., 2005; Carpenter et al., 2008) and less cooperatively (Egas and Riedl, 2008; Burks et al., 2009). Other groups of people may be more generous to the poor and more likely to accept unfair offers from proposers with low objective wealth. Thirdly, college students usually come from a quite narrow age range and are concentrated at the upper levels of educational background. Age and education are the two most powerful demographic factors influencing attitudes and attitudinal processes (Sears, 1986). Future studies are suggested to collect data from more general populations. This allows researchers to test whether different sub-samples exhibit different behavioral and electrophysiological patterns. Furthermore, cultural differences may also play an essential role. With the economic boom in China, Chinese citizens have not benefited evenly from economic growth, and the gap between the rich and the poor has been widening. Because of the enormous financial inequity arising between citizens, dissociation between explicit and implicit attitudes towards the rich in China has been reported. The explicit attitude towards the rich was negative, but the implicit attitude was positive (Zhou and Wang, 2007). That is, though the wealth gap leads to negative feelings toward the rich, strong motivations of becoming one of the wealthy few can be easily traced in Chinese society. The conflicting attitudes toward the rich seem quite complex, and this social and cultural background may also play a role in fairness perception. Similar research should be conducted in other countries with varying economic status. Cross-cultural studies may provide new insights into this phenomenon. What’s more, the future study may consider the influence of stake size. Issues concerning stake size have long been recognized as a challenge for the external validity not only of the current study, but for all findings obtained within the UG (for a review, see Hoffman et al., 1996). In UG task, participants have to make tradeoff between self-interest and fairness perception. The effect of stake size may be related to the relative impact of the unfair offer. In present study, the stake size is relatively small. When the stake size is larger, rejections may lead to a higher cost of self-interest. In view of personal loss, participants may be more likely to accept the unfair offers from proposers with high objective wealth. Thus, the modulation effect by objective wealth may be weakened.

As mentioned above, the factors such as cultural differences, the stake size and participants’ incomes may have significant impacts on the participants’ decision making. For future research, there is a fundamental question that needs to be described clearly in the first, i.e., what are the participants’ fairness principles when they make decisions? Afterward, the research model may be well designed to make a study of the related influence factors. It is worth mentioning that, the different influence factors may be interactive, nonlinear and dynamic. It is necessary to explore their working process and systematic mechanism with the help of artificial intelligence and system simulation. Furthermore, although this study provided new ERPs evidences, analyzing behavior and linking it to the neural signal can be extremely helpful. Multimodal data provide complementary information to each other, thus helping produce more robust predictions and explanations. In future research, self-reported data of expectations and fairness principles should be involved. Besides, response times are almost always reported in studies using the ultimatum game paradigm. It would be interesting to analyze and report them, which can give information concerning the decision-making process.

## Conclusion

The present study explored how perceived objective wealth would modulate fairness-related decision making. With respect to modulation of responses to unfairness by objective wealth, people tended to reject unfair offers from proposers with high (relative to low) objective wealth. Focusing on modulation of perception of unfairness by objective wealth, enhanced FRN amplitude was induced when receiving unfair offers from proposers with high objective wealth compared with proposers with low objective wealth. Moreover, the LFN amplitude was larger for all the conditions which are not high-fair, which might be the only option that did not elicit any ambiguity. To conclude, both perception of fairness and responses to it were behaviorally and neurally modulated by objective wealth.

## Data Availability Statement

The raw data supporting the conclusions of this article will be made available by the authors, without undue reservation.

## Ethics Statement

The studies involving human participants were reviewed and approved by the Ethical Committee of Ningbo University. The participants provided their written informed consent to participate in this study.

## Author Contributions

GP and JJ conceived and designed the experiments. GP, JJ, and CF performed the experiments and analyzed the data. GP, JJ, and TL wrote the manuscript. All authors contributed to the article and approved the submitted version.

## Conflict of Interest

CF was employed by company Zhejiang Radio and TV Group. The remaining authors declare that the research was conducted in the absence of any commercial or financial relationships that could be construed as a potential conflict of interest.
